# Telomere length predicts for outcome to FCR chemotherapy in CLL

**DOI:** 10.1038/s41375-019-0389-9

**Published:** 2019-01-30

**Authors:** Kevin Norris, Peter Hillmen, Andrew Rawstron, Robert Hills, Duncan M. Baird, Christopher D. Fegan, Chris Pepper

**Affiliations:** 1 Division of Cancer & Genetics, Cardiff University, School of Medicine, Heath Park, Cardiff, UK; 2 Section of Experimental Haematology, Leeds Institute of Cancer and Pathology (LICAP), University of Leeds, Leeds, UK; 3 Nuffield Department of Population Health, University of Oxford, Richard Doll Building, Old Road Campus, Roosevelt Drive, Oxford, OX3 7LF USA; 4 University of Sussex, Brighton and Sussex Medical School, Brighton, UK

**Keywords:** Cancer genetics, Translational research

## Abstract

We have previously shown that dividing patients with CLL into those with telomeres inside the fusogenic range (TL-IFR) and outside the fusogenic range (TL-OFR) is powerful prognostic tool. Here, we used a high-throughput version of the assay (HT-STELA) to establish whether telomere length could predict for outcome to fludarabine, cyclophosphamide, rituximab (FCR)-based treatment using samples collected from two concurrent phase II studies, ARCTIC and ADMIRE (*n* = 260). In univariate analysis, patients with TL-IFR had reduced progression-free survival (PFS) (*P* < 0.0001; HR = 2.17) and shorter overall survival (OS) (*P* = 0.0002; HR = 2.44). Bifurcation of the *IGHV*-mutated and unmutated subsets according to telomere length revealed that patients with TL-IFR in each subset had shorter PFS (HR = 4.35 and HR = 1.48, respectively) and shorter OS (HR = 3.81 and HR = 2.18, respectively). In addition, the OS of the TL-OFR and TL-IFR subsets were not significantly altered by *IGHV* mutation status (*P* = 0.61; HR = 1.24 and *P* = 0.41; HR = 1.47, respectively). In multivariate modeling, telomere length was the dominant co-variable for PFS (*P* = 0.0002; HR = 1.85) and OS (*P* = 0.05; HR = 1.61). Taken together, our data suggest that HT-STELA is a powerful predictor of outcome to FCR-based treatment and could be used to inform the design of future risk-adapted clinical trials.

## Introduction

The management of chronic lymphocytic leukemia (CLL) has undergone a dramatic change in the last decade due to the introduction of effective new therapeutic regimens. However, despite the demonstrable efficacy of B-cell receptor signaling antagonists like ibrutinib [1, 2], the gold standard treatment for fit patients with CLL is still fludarabine, cyclophosphamide, rituximab (FCR) combination chemoimmunotherapy [3–7]. However, longer follow-up studies have shown that not all patients benefit equally from FCR; *IGHV*-mutated patients have a significantly better overall survival compared to *IGHV-*unmutated patients [5, 8–10] and patients with 17p abnormalities show markedly inferior outcomes with this regimen [8–10].

We and others have previously shown that telomeres play a pivotal role in the pathogenic progression and outcome of CLL, with telomere length analysis providing independent prognostication in all stages of CLL [11–14]. Using high-resolution telomere length analysis (STELA), we were able to show that patients with very short, dysfunctional telomeres are prone to chromosome fusion events; these patients commonly demonstrate increased genomic complexity [11, 14]. Furthermore, many of the losses and gains of genetic material observed in these patients appear to be focused at chromosome-ends and are consistent with the types of genetic rearrangements that can occur because of telomere fusion, leading to the initiation of cycles of anaphase-bridging, breakage, and fusion [15]. Importantly, we have shown previously that telomere length is the strongest determinant of clinical outcome in CLL and predicts for survival following chemotherapy [16, 17].

Here, we investigated telomere length in the context of frontline treatment with fludarabine, cyclophosphamide, rituximab (FCR)-based regimens. We considered whether telomere length was predictive of clinical outcome, using high-throughput single telomere length analysis (HT-STELA), in 275 samples from two concurrent UK trials of FCR-based treatment, ARCTIC and ADMIRE. We then compared the predictive power of HT-STELA with the commonly used prognostic markers: β2 microglobulin (β2M), fluorescence in situ hybridization (FISH) cytogenetics, CD38 expression, ZAP70 expression and *IGHV* mutation status.

## Materials and methods

All the clinical samples used were taken at study entry and were obtained from the Bloodwise funded UK CLL Trials Biobank, University of Liverpool. Of the 260 evaluable samples for telomere length, 128 were derived from the ARCTIC trial; 64 patients were randomized to receive standard dose fludarabine, cyclophosphamide and rituximab (FCR) and 64 received fludarabine, cyclophosphamide, mitoxantrone, and mini rituximab (FCM-mini R). One-hundred thirty-two samples were evaluated from the the ADMIRE study; 64 patients were randomized to receive standard dose FCR and 68 received fludarabine, cyclophosphamide, mitoxantrone and rituximab (FCMR). 17p mutation or deletion were exclusion criteria from both of these studies due to their association with poor outcome following FCR treatment [18, 19]. However, due to the lag time in genetic analysis, it was later established that 16 patients with a 17p deletion were enrolled in the trials. The median follow-up in the combined cohort was 4 years and there were 51 deaths at the censor point. The demographics of the cohort are summarized in Table 1. Due to the study inclusion criteria for ARCTIC and ADMIRE, disease burden was generally high with a mean absolute lymphocyte count of 87.6 × 10^6^/mL (range 3.3–547.5). However, to avoid potential measurement error caused by the presence of non-malignant cell fractions, telomere length was assessed on DNA extracted from purified CD19^+^ B-cells using a B-cell isolation kit (Miltenyi Biotec) using an adaptation of chromosome-specific STELA to allow for high-throughput analysis (HT-STELA). Briefly, the previously published STELA protocol [20, 21] was adapted to use telomere-adjacent primers specific for the XpYp telomere (XpYpC: 5ʹ-CAGGGACCGGGACAAATAGAC-3ʹ) and the 7q telomere (7qK1: 5ʹ-GGGCACTGCCTCGCTTTGA-3ʹ), in triplicate 30 μL PCR reactions each containing 30 ng of genomic DNA. Thermal cycling conditions were: 23 cycles of 94 °C for 20 s, 65 °C for 30 s, and 68 °C for 5 min. Amplified fragments were resolved using capillary gel electrophoresis and mean telomere length determined using PROSize software (AATI, Ankeny, Iowa, USA). Patients were bifurcated using the previously determined mean XpYp telomere length threshold for telomere dysfunction [17], creating two patient groups: one with telomere lengths equal or less than the mean of the fusogenic range; inside the fusogenic range (TL-IFR) and the other with telomere lengths greater than the mean of the fusogenic range; outside the fusogenic range (TL-OFR). The numerical threshold that defined these two groups using XpYp telomere analysis was subsequently adjusted for the 7q telomere according to the *y* *=* *mx* *+* *c* regression line generated by plotting XpYp telomere length against 7q telomere length. 7q HT-STELA was used in preference to XpYp HT-STELA as a larger subset of CLL samples failed to amplify the XpYp telomere (24/275) when compared with the 7q telomere (15/275). For consistency, all of the subsequent analyses were carried out on the data generated using 7q HT-STELA (*n* = 260). However, it should be noted that there was a strong correlation between the telomere lengths at each chromosome end (*P* < 0.0001; *r*^2^ = 0.72). In univariate analyses, all the individual prognostic markers were considered as categorical variables using previously established thresholds. In multivariate modeling, the same parameters were evaluated as both categorical and continuous variables. The variables included in the model were age, cytogenetic groups (del 11q and del 17p), *IGHV* mutation status, CD38 expression, ZAP70 expression, β2M, absolute lymphocyte count, telomere length. Statistical analysis was carried out using Prism 6.0 (Graphpad Software Inc., La Jolla, CA, USA) and SAS version 9.3 software (SAS Institute, Cary, NC, USA). Univariate comparisons for progression-free survival (PFS) and overall survival (OS) were conducted with the logrank test and displayed as Kaplan-Meier curves. Multivariate analyses were performed using a Cox proportional hazard model with forward selection. In all cases *P* < 0.05 was considered significant.

**Table 1:** Summary of patient characteristics for the cohort of 260 patients on which 7q HT-STELA was performed

Parameter	Number of patients
ARCTIC	128
FCM-mini R	64
FCR	64
ADMIRE	132
FCMR	68
FCR	64
Median ALC (x10^6^/mL)	87.6
Median time to progression (months)	42.3
Median follow-up (months)	47.2
*IGHV*-M	106
*IGHV*-UM	148
ND	6
CD38^−^( < 20%)	157
CD38^+^ ( ≥ 20%)	102
ND	1
β2 M ( < 3.5 mg/L)	65
β2 M (≥3.5 mg/L)	176
ND	19
11q-	50
17p-	16
Other FISH cytogenetics	194
7q telomere analysis	
IFR	83
OFR	177

ARCTIC: randomized phase IIB trial of fludarabine, cyclophosphamide and rituximab versus fludarbine, cyclophosphamide, Mitoxantrone with low-dose rituximab in previously untreated CLL patients

ADMIRE: randomized phase IIB trial of fludarabine, cyclophosphamide and rituximuab versus fludarabine, cyclophosphamide, mitoxantrone and rituximuab in previously untreated CLL

*IGHV*-M: mutated *IGHV* genes; ≥2% deviation from the germline immunoglobulin sequence

*IGHV*-UM: unmutated *IGHV* genes; < 2% deviation from the germline immunoglobulin sequence

β2M: β2 microglobulin

11q^-^: mutations or deletions in the long arm of chromosome 11

17p^-^: mutations or deletions in the short arm of chromosome 17

7q telomere analysis–IFR; ≤ the mean telomere length of the fusogenic range, *OFR* outside the mean telomere length of the fusogenic range

*ND* not determined, *ALC* absolute lymphocyte count

## Results

### High-throughtput STELA allows for the reliable and rapid evaluation of telomere length in CLL

**Fig. 1:**
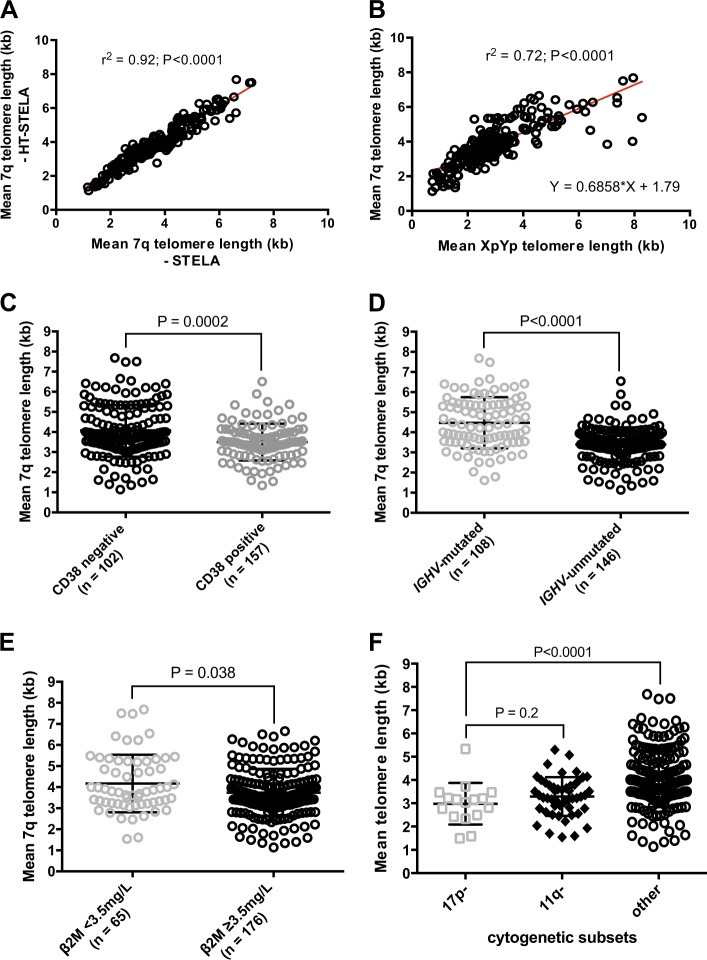
Telomere length measurements using HT-STELA and the relationship between telomere length and other prognostic markers. HT-STELA was developed to overcome some of the technical complexities of the original STELA assay and to adapt the assay to make it suitable for high-throughput analysis. **a** shows the strong correlation between original STELA and HT-STELA. **b** shows the strong correlation between telomere length measured using HT-STELA with probes specific for the XpYp and 7q telomeres. **c**–**f** 7q HT-STELA confirmed that poor prognostic subsets all had significantly shorter telomere length profiles than their respective good prognosis counter-parts

Our previously described single molecule STELA assay is both technically challenging and time consuming making it unsuitable for the evaluation of large numbers of samples [11]. To overcome these problems, we developed a modification of the STELA assay to facilitate the high-throughput evaluation of samples (HT-STELA). Here we present the first evidence that this technique is comparable to standard STELA and can be used to quickly and reliably predict for outcome following FCR-based therapy in samples derived from two UK CLL trials, ARCTIC and ADMIRE [6, 7]. To evaluate the utility of HT-STELA for the analysis of telomere length in CLL, we undertook a comparison of both STELA and HT-STELA on 260 patient samples, at two separate chromosome-ends using primers designed to specifically amplify the XpYp and the 7q telomeres. We showed strong concordance between the STELA and HT-STELA assays; Fig. 1a shows 7q STELA versus 7q HT-STELA (*r*^2^ = 0.92, *P* < 0.0001). In addition, Fig. 1b shows the correlation between the XpYp telomere length and the 7q telomere length using HT-STELA (*r*^2^ = 0.72, *P* < 0.0001). It is worthy of note that the variance between the two chromosome-ends was most apparent in samples with long telomeres and no sample changed category (TL-IFR, TL-OFR) as a consequence of measuring a different telomere. Consistent with previous reports [11, 14, 15, 21], short telomere length was significantly associated with CD38 positivity (*P* = 0.0002, Fig. 1c) and elevated β2M (*P* = 0.038, Fig. 1d), *IGHV*-unmutated cases (*P* < 0.0001, Fig. 1e) and high-risk cytogenetic lesions (*P* < 0.0001, Fig. 1f).

### Telomere length predicts for progression-free and overall survival

**Fig. 2:**
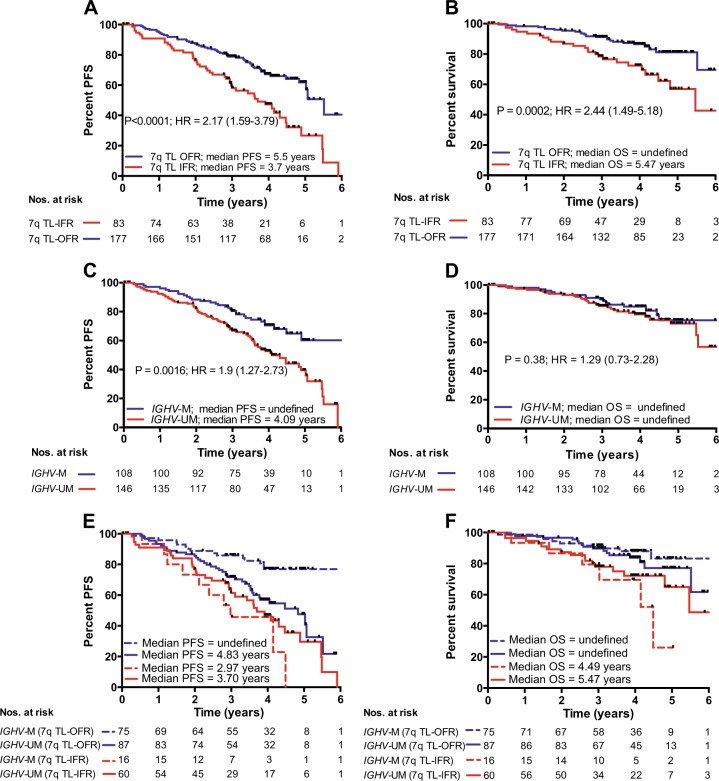
Stratification of patients by telomere length predicts for PFS and OS following FCR-based treatment. Bifurcation of the patient cohort according to the previously defined telomere length threshold for telomere dysfunction was predictive of (**a**) PFS and (**b**) OS. Patients whose telomere length were ≤ the mean of the fusogenic range (TL-IFR) showed shorter PFS and OS than those patients with mean telomere length outside of the fusogenic range (TL-OFR). Comparison of the same cohort based on *IGHV* mutation status showed that *IGHV*-unmutated patients had **c** a shorter median PFS but no difference in **d** OS when compared with *IGHV*-mutated patients. *IGHV*-mutated and *IGHV*-unmutated groups could be divided according to telomere length, which identified distinct subsets in each group with **e** different PFS and **f** different OS

We first set out to establish whether telomere length could predict for outcome to FCR-based therapy in the entire cohort of samples, regardless of randomization. The cohort was split according to our previously defined threshold of telomere dysfunction; telomere lengths equal or less than the mean of the fusogenic range (TL-IFR) and telomere lengths greater than the mean of the fusogenic range (TL-OFR). Patients with TL-IFR showed significantly shorter PFS (*P* < 0.0001; Fig. 2a) and reduced OS (*P* = 0.0002; Fig. 2b). In the same cohort, *IGHV* mutation status was predictive of PFS (*P* = 0.0016; Fig. 2c) but not OS (*P* = 0.38; Fig. 2d). Bifurcation of the *IGHV*-mutated and *IGHV*-unmutated groups into TL-IFR and TL-OFR subsets showed that telomere length identified patients with different PFS (Fig. 2e) and different OS (Fig. 2f). Neither CD38 expression (< / > 20%) nor β2M (< / > 3.5 mg/L) expression were predictive of PFS or OS (Supplementary Fig. 1A–D). In addition, removal of the 17p deleted/mutated patients from the analysis (*n* = 16) did not significantly alter the predictive power of telomere length in terms of PFS and OS (Supplementary Fig. 2). Although the 7q HT-STELA analysis was selected for use in this study, XpYp HT-STELA showed similar predictive power for both PFS and OS (Supplementary Fig. 3).

### Telomere length identifies differential outcomes in *IGHV*-mutated and *IGHV*-unmutated groups

**Fig. 3:**
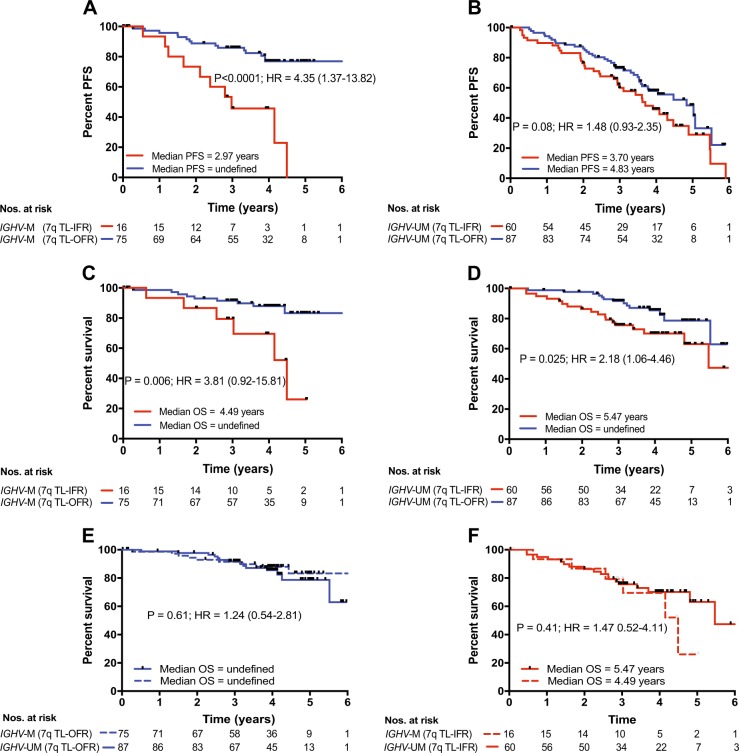
The impact of telomere length in *IGHV*-mutated and *IGHV*-unmutated groups. Dividing the *IGHV*-mutated and *IGHV*-unmutated prognostic groups according to telomere length revealed **a** a significant difference in PFS in the *IGHV*-mutated group with short telomere patients having shorter PFS. **b** Short telomere length in the *IGHV*-unmutated group showed a trend towards reduced PFS but this was not significant. In terms of OS, the short telomere subsets in both **c** *IGHV*-mutated and **d** *IGHV*-unmutated groups showed significantly reduced survival. The OS of patients with **e** long telomeres (TL-OFR) and **f** short telomeres (TL-IFR) were not significantly different regardless of *IGHV* mutation status

A number of studies, including the German CLL8 study, have shown that a proportion of *IGHV*-mutated patients, treated with FCR, can achieve long-term remissions [8–10]. Here, we show that patients with *IGHV*-mutated CLL with short telomeres (TL-IFR) were more likely to progress during the follow-up period (*P* < 0.0001, HR = 4.35; Fig. 3a) and more likely to succumb to their disease (*P* = 0.006, HR = 3.81; Fig. 3c) than patients with TL-OFR. In the *IGHV*-unmutated group, most patients showed clinical progression during the follow-up period; the median PFS of of the TL-IFR subset was 3.7 years versus 4.83 years in the TL-OFR subset (*P* = 0.08, HR = 1.48; Fig. 3b). The *IGHV*-unmutated TL-IFR subset showed a significantly worse OS when compared with the TL-OFR subset (*P* = 0.025, HR = 2.18; Fig. 3d). It is of interest that the *IGHV*-mutated subset with TL-IFR had a median OS almost one year less than the *IGHV*-unmutated subset with TL-IFR (4.49 years versus 5.47 years). Although the number of *IGHV*-mutated patients with TL-IFR was relatively small (*n* = 16), our data suggests that telomere length can identify a subset of “bad risk” *IGHV*-mutated patients who do not respond well to FCR. Furthermore, splitting the cohort into TL-IFR and TL-OFR subsets was a more powerful determinant of clinical outcome than *IGHV* mutation status. Indeed, the OS of patients with TL-OFR and TL-IFR were not significantly different regardless of *IGHV* mutation status (Fig. 3e, f respectively). It is worthy of note that of the 16 “bad risk” *IGHV*-mutated patients only 2/16 used the *IGHV* 3–21 gene. In terms of other known prognostic factors, 5/16 were 11q-, 2/16 were 17p-, 10/16 were CD38^+^ and 11/16 had β2M ≥3.5 mg/L.

### Telomere length is predictive in both ARCTIC and ADMIRE regardless of randomization

**Fig. 4:**
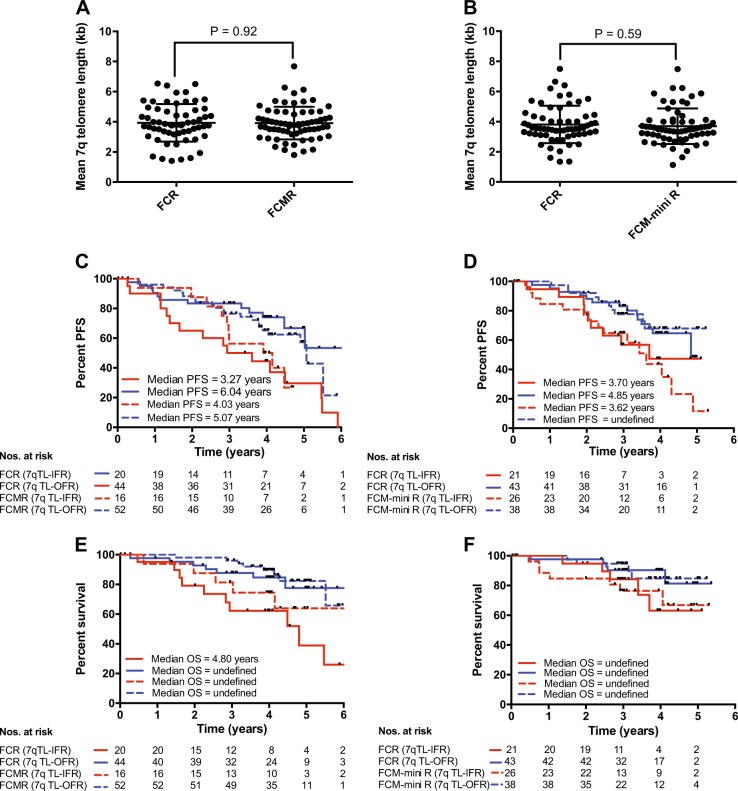
Comparison of telomere length in patients treated in ADMIRE and ARCTIC. The mean telomere length were not significantly different between patients randomized in **a** ADMIRE and **b** ARCTIC clinical trials. Stratification accord to telomere length revealed differential PFS (**c** and **d**) and OS (**e** and **f**) in both clinical trials regardless of randomization

We next analyzed the effect of telomere length in the samples derived from the two separate trials and between the randomization arms of the two studies. We showed that there was no difference in telomere length between patients randomized to receive FCR and FCMR in the ADMIRE study (*P* = 0.92; Fig. 4a) and those randomized to receive FCR and FCM-mini R in the ARCTIC study (*P* = 0.59; Fig. 4b). In both studies, telomere length analysis was able to identify subsets of patients with different PFS (Fig. 4c, d) and different OS (Fig. 4e, f) to the FCR-based treatments given.

### Telomere length is predictive in patients treated with standard dose FCR

**Fig. 5:**
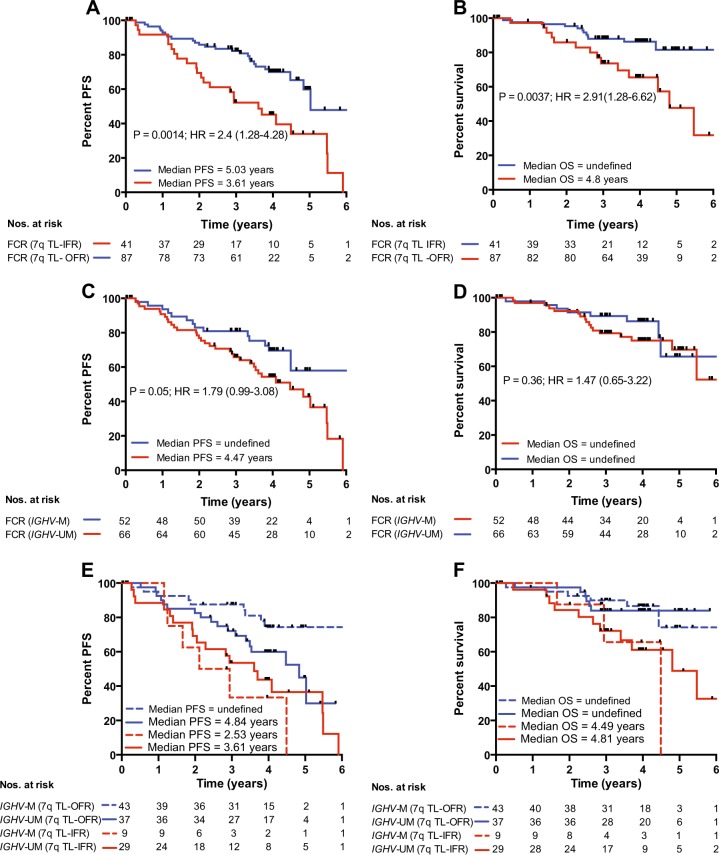
The impact of telomere length in FCR-treated patients derived from ADMIRE and ARCTIC. Analysis of the patient cohort treated with standard dose FCR demonstrated that telomere length was a strong predictor of **a** PFS and **b** OS. Comparison of the effect of *IGHV* mutation status in the same cohort showed it was **c** predictive of PFS but **d** was not predictive of OS. Bifurcation of the *IGHV*-mutated and *IGHV*-unmutated groups accord to telomere length again reveals subsets with distinct **e** PFS and **f** OS

Although we demonstrated that mean telomere length was not different between the patients randomized within both ADMIRE and ARCTIC, the published data on the two trials suggested a randomization effect [6, 7] so we performed a sub-analysis of the effect of telomere length in all patients treated with standard dose FCR (*n* = 136). Patients with short telomeres who received FCR showed a significantly shorter PFS (*P* = 0.0014, HR = 2.4; Fig. 5a) and shorter OS (*P* = 0.0037, HR = 2.91; Fig. 5b). In the same cohort, *IGHV* mutation status was predictive of PFS (*P* = 0.05, HR = 1.79; Fig. 5c) but not OS (*P* = 0.36, HR = 1.47; Fig. 5d). Once again, telomere length was able to identify a subset of both *IGHV*-mutated and *IGHV*-unmutated cases with a differential PFS and OS when treated with FCR (Fig. 5e, f respectively). Consistent with the analysis of the entire cohort, the *IGHV*-mutated cases with TL-IFR showed a shorter median PFS (2.53 years) and a shorter median OS (4.49 years) than the *IGHV*-unmutated cases with TL-IFR (3.61 years and 4.81 years respectively; Supplementary Fig. 4).

### Telomere length is the dominant co-variable in multivariate analysis

In univariate analysis we have shown that telomere length is a strong predictor of PFS and OS in FCR-based clinical trials. However, given the association between short telomeres and other high-risk prognostic markers, we next set out to determine whether telomere length would retain predictive significance in multivariate analysis using a Cox proportional hazard model with forward selection. Table 2A and  2B summarize the univariate and multivariate analysis for PFS and OS; telomere length was the dominant co-variable with HR = 1.85 (1.33–2.28) and HR = 1.61 (0.99–2.63) respectively. Indeed, the inclusion of telomere length in the model rendered all of the other markers insignificant even when the inclusion threshold was reduced to *P* = 0.1. The tables show the adjusted *P*-values for telomere length when all of the other parameters were forced into the model and the unadjusted *P*-values for each parameter without forcing. In keeping with the previously published data from the ARCTIC and ADMIRE studies [6, 7], age was not an independent prognostic factor in either univariate or multivariate analysis.

**Table 2A:** Univariate and multivariate analysis for time to progression

	Univariate HR (95% CI)	*P*-value	Multivariate HR (95% CI)	*P*-value	Straight selection (no forcing)
B2M	0.99 (0.68–1.47)	0.7	1.03 (0.61–1.72)	**	*P* = 0.98
Lymphocyte count	1.00 (1.00–1.00)	0.07	1.00 (0.998–1.003)	**	*P* = 0.5
*IGHV* mutation status	1.90 (1.27–2.73)	0.0016	1.08 (0.64–1.84)	**	*P* = 0.8
Del 11q23	1.49 (0.97–2.27)	0.07	1.56 (0.93–2.56)	**	*P* = 0.14
CD38	1.04 (0.67–1.59)	0.99	1.40 (0.86–2.27)	**	*P* = 0.4
7q TL	2.17 (1.59–3.79)	<0.0001	1.79 (1.25–2.56)	0.002	1.85 (1.33–2.28) *P* = 0.0002

**Table 2B:** Univariate and multivariate analysis for overall survival

	Univariate HR (95% CI)	*P*-value	Multivariate HR (95% CI)	*P*-value	Straight selection (no forcing)
B2M	1.18 (0.62–2.19)	0.63	1.25 (0.69–1.97)	**	*P* = 0.3
Lymphocyte count	0.99 (0.99–1.01)	0.2	0.99 (0.99–1.001)	**	*P* = 0.3
*IGHV* mutation status	1.29 (0.73–2.28)	0.38	1.33 (0.65–2.70)	**	*P* = 0.3
Del 11q23	1.05 (0.52–2.10)	0.9	1.09 (0.48–2.43)	**	*P* = 0.7
CD38 status	1.13 (0.65–1.98)	0.66	1.33 (0.63–2.78)	**	*P* = 0.5
7q TL	2.44 (1.49–5.18)	0.0002	1.81 (1.08–3.13)	0.02	1.61 (0.99–2.63) *P* = 0.05

β2M: β2 microglobulin expression (≥3.5 mg/L versus < 3.5 mg/L)

*IGHV* mutation status: *IGHV*-unmutated versus *IGHV*-mutated

Del 11q23: patients with deletions in chromosome 11q23 versus those without

CD38: CD38 expression (≥20% expression versus < 20% expression)

7q TL: Telomere length measured at chromosome 7q

** indicates forced into multivariable analysis (i.e., analyses adjusted for these factors)

## Discussion

It has been previously shown that a proportion of chronic lymphocytic leukemia (CLL) patients have critically short telomeres and this is associated with genomic instability and inferior clinical outcome [11, 14]. Indeed, telomere dysfunction was shown to be the most powerful predictor of survival in a cohort of 321 Patients with CLL and allowed the accurate stratification of Binet stage A patients into those with indolent disease and those with poor prognosis [17]. Furthermore, patients with long telomeres showed superior prognosis regardless of their *IGHV* mutation status CD38 expression, ZAP70 expression or cytogenetic risk group. In keeping with this finding, telomere dysfunction was the dominant variable in multivariate analysis. More recently we proved that telomere dysfunction predicted for response to the combination of fludarabine and cyclophosphamide in the UK CLL4 clinical trial [16]. Here, we describe for the first time the utility of a high-throughput adaptation of single telomere length analysis (HT-STELA), in 260 evaluable samples derived from the ARCTIC and ADMIRE clinical trials; both trials evaluated the chemoimmunotherapy combination of fludarabine, cyclophosphamide, rituximab (FCR) in previously untreated Patients with CLL.

We showed that HT-STELA could be reliably used to determine telomere length at two different chromosome-ends. Our data also confirmed our previous findings that telomere length measurement at any chromosome end is a bellwether of the telomere length of all chromosome-ends in the absecnce of genetic lesions that lead to the loss of telomeric material. Shorter telomere length was associated CD38 positivity, increased β2 microglobulin, *IGHV*-unmutated gene sequences and high-risk cytogenetics. Division of the cohort according to the previously determined mean telomere length threshold for telomere dysfunction [18] revealed that telomere length is a powerful predictor of both PFS and OS in patients treated with FCR-based therapies. In contrast, CD38 expression and β2 microglobulin expression were not predictive and *IGHV* mutation status was only predictive of PFS. It remains unclear why our findings are somewhat different to the German CLL8 study in terms of OS but the longer median follow-up in the CLL8 study (5.9 years versus 4 years) may be a contributing factor as there appears to be a steepening in the FCR-treated *IGHV*-unmutated survival curve after 5 years [8]. In any case, telomere length is able to identify good risk and bad risk patients in both the *IGHV*-mutated and *IGHV*-unmutated groups (Figs. 2e, f and  3). Furthermore, telomere length appears to identify a subset of *IGHV*-mutated cases with a particularly poor response to FCR-based treatment; these patients had a worse median PFS and median OS than their *IGHV*-unmutated counter-parts. Furthermore, the adverse impact of short telomeres could not be explained by *IGHV* gene usage (*IGHV* 3–21) [22] or high-risk cytogenetic lesions (11q-/17p-). Although this finding is potentially important, it would need to be confirmed in a larger study as the number of *IGHV*-mutated cases with short telomeres was small (*n* = 16) when compared with the *IGHV*-unmutated subset (*n* = 60). It is of particular note that telomere length appears to be a critical determinant of clinical outcome and superseded the prognostic impact of *IGHV* mutation status in this cohort of patients (Fig. 3e, f).

Since the samples analyzed in this study were drawn from two separate clinical trials, ADMIRE and ARCTIC, we next evaluated the influence of telomere length in each trial separately and within each of the respective trial arms. There was no significant difference in mean telomere in either arm of the trials but telomere length was able to identify good risk and bad risk groups regardless of randomization (Fig. 4). Subsequent analysis of only the FCR-treated samples drawn from both trials (*n* = 136) revealed a similar picture to the entire cohort analysis. Telomere length was predictive of PFS and OS and could bifurcate *IGHV*-mutated and *IGHV*-unmutated groups in terms of PFS and OS (Fig. 5 and Supplementary Fig. 2).

Finally, we undertook multivariate modeling to establish the predictive hierarchy of the prognostic factors measured in this study. Using a forward selection model, telomere length was shown to be the dominant co-variable for both PFS and OS (Table 2A and B, respectively). The strong correlation between short telomeres and other poor-risk prognostic tools, including *IGHV* mutation status, appeared to render these markers insignificant when telomere length was included. Given the relative speed and simplicity of HT-STELA, our data suggest that it can reliably identify patients who will benefit from FCR treatment and those who will not. It remains to be established whether HT-STELA can also be used to predict for response to non-chemotherapeutic agents like ibrutinib but it seems likely that adoption of this predictive biomarker into clinical trials design could enable rational, risk-adapted, trials with the aim of treating all patients with the optimal therapeutic regimen.

## Supplementary information

Supplementary Figure 1

Supplementary Figure 2

Supplementary Figure 3

Supplementary Figure 4
