# Supplementary material for: Telomere length predicts for outcome to FCR chemotherapy in CLL
Source: Leukemia. 2019 Jan 30;33(8):1953–63. doi: 10.1038/s41375-019-0389-9 (PMC6756045; doi:10.1038/s41375-019-0389-9)
Supplement: Supplementary file 1 — Supplementary Figure 1 [file 41375_2019_389_MOESM1_ESM.pdf]

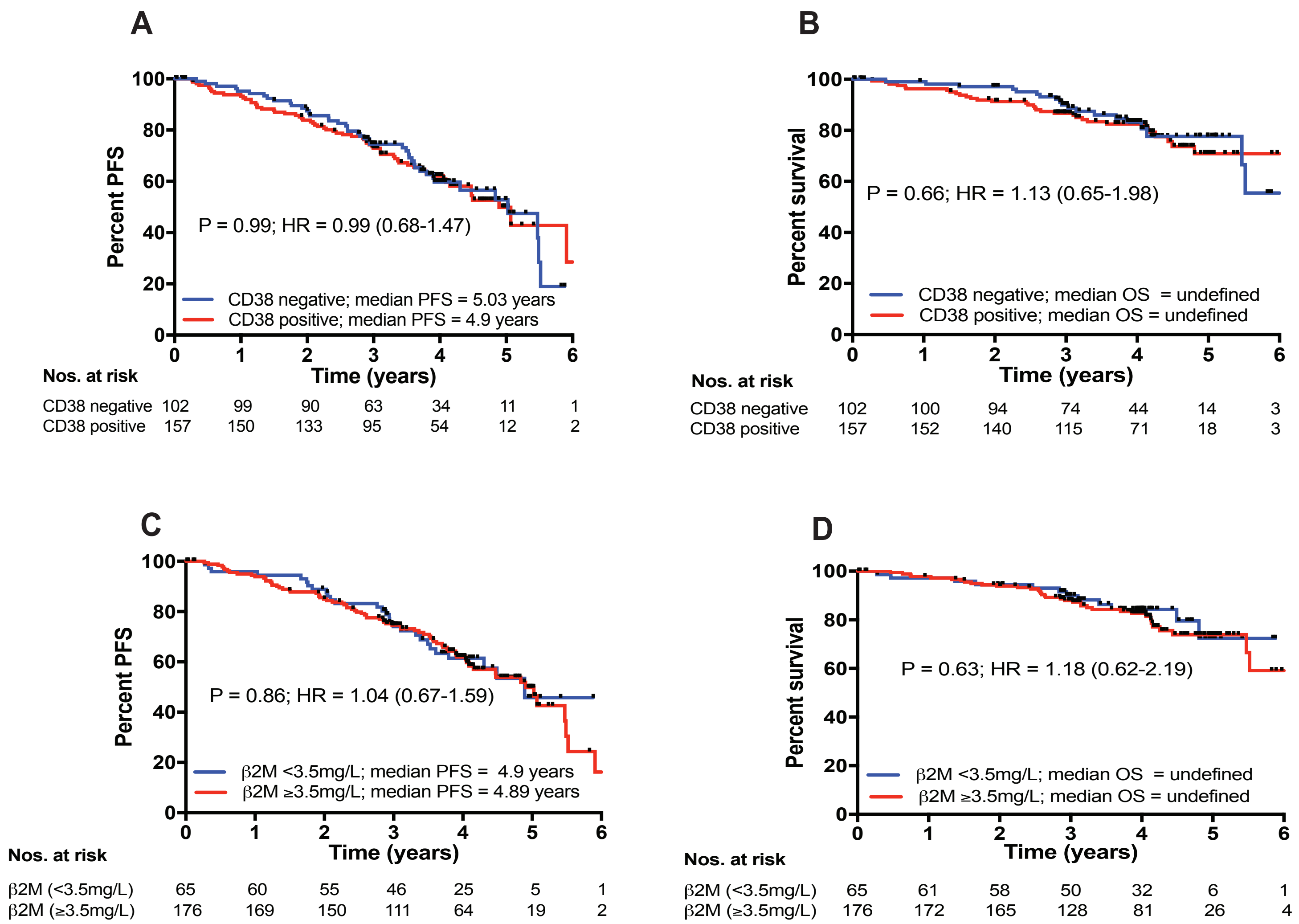

Supplementary Figure 1. CD38 expression and β2 microglobulin levels do not predict for response to FCR. CD38 expression (A and B) and β2 microglobulin levels (C and D) were not predictive of PFS or OS in patients treated with FCR-based therapies.
