# Supplementary material for: Telomere length predicts for outcome to FCR chemotherapy in CLL
Source: Leukemia. 2019 Jan 30;33(8):1953–63. doi: 10.1038/s41375-019-0389-9 (PMC6756045; doi:10.1038/s41375-019-0389-9)
Supplement: Supplementary file 2 — Supplementary Figure 2 [file 41375_2019_389_MOESM2_ESM.pdf]

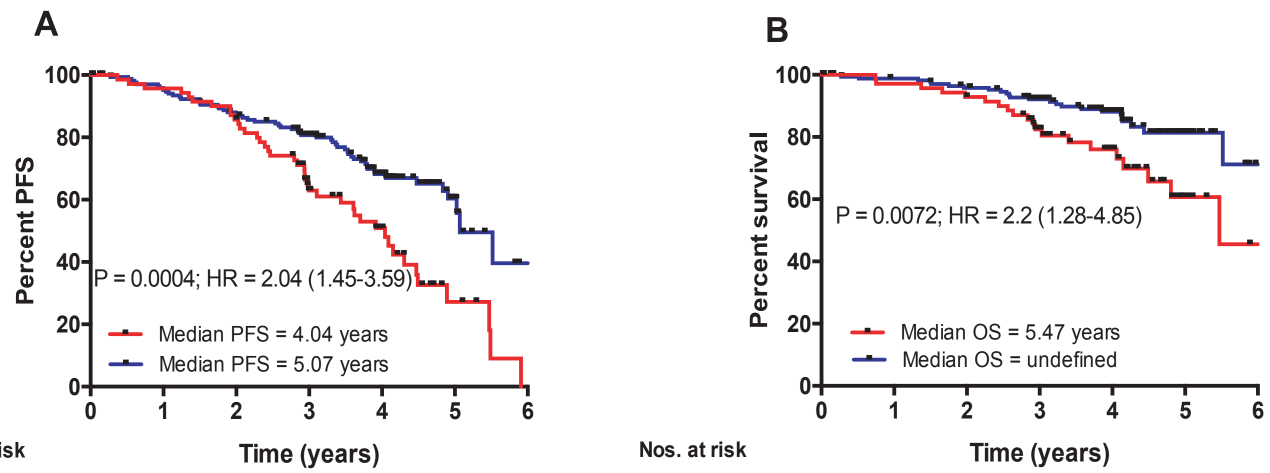

Nos. at risk (A)

	0	1	2	3	4	5	6
7q TL IFR	71	68	60	36	20	6	1
7q TL OFR	172	161	146	116	69	16	2

Nos. at risk (B)

	0	1	2	3	4	5	6
7q TL IFR	71	69	65	45	28	8	3
7q TL OFR	172	166	159	129	84	24	2

Supplementary Figure 2. The impact of telomere length in the entire cohort following exclusion of patients with 17p deletion/mutation. Telomere length was predictive of (A) PFS and (B) OS with and without the inclusion of patients with 17p deletion/mutation (n = 16).
