# Supplementary material for: Telomere length predicts for outcome to FCR chemotherapy in CLL
Source: Leukemia. 2019 Jan 30;33(8):1953–63. doi: 10.1038/s41375-019-0389-9 (PMC6756045; doi:10.1038/s41375-019-0389-9)
Supplement: Supplementary file 3 — Supplementary Figure 3 [file 41375_2019_389_MOESM3_ESM.pdf]

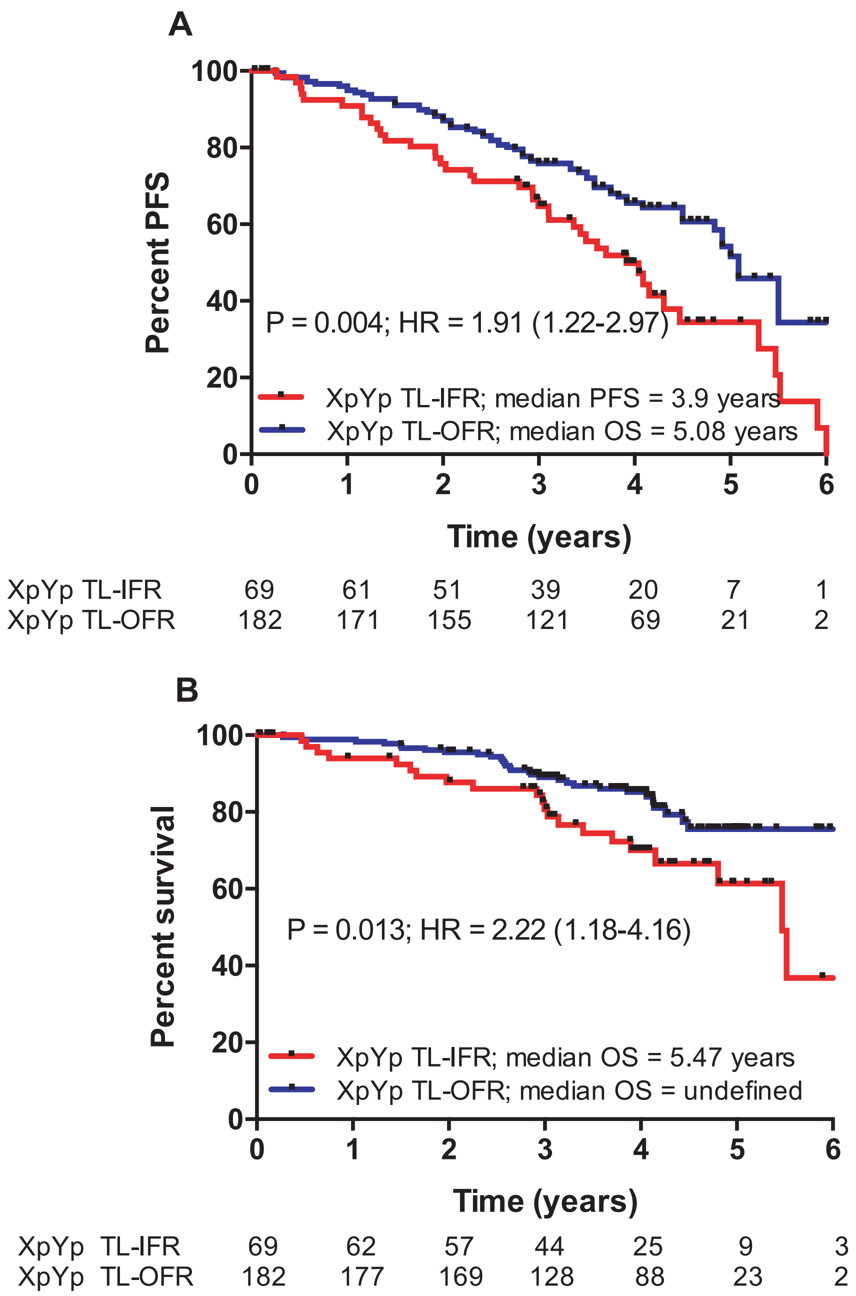

Supplementary Figure 3. Stratification of patients by telomere length predicts for PFS and OS following FCR-based treatment. Bifurcation of the patient cohort according to the previously defined telomere length threshold for telomere dysfunction was predictive of (A) PFS and (B) OS. Patients whose telomere length were inside the fusogenic range (TL-IFR) showed shorter PFS and OS than those patients with mean telomere length outside of the fusogenic range (TL-OFR).
