# Supplementary material for: Telomere length predicts for outcome to FCR chemotherapy in CLL
Source: Leukemia. 2019 Jan 30;33(8):1953–63. doi: 10.1038/s41375-019-0389-9 (PMC6756045; doi:10.1038/s41375-019-0389-9)
Supplement: Supplementary file 4 — Supplementary Figure 4 [file 41375_2019_389_MOESM4_ESM.pdf]

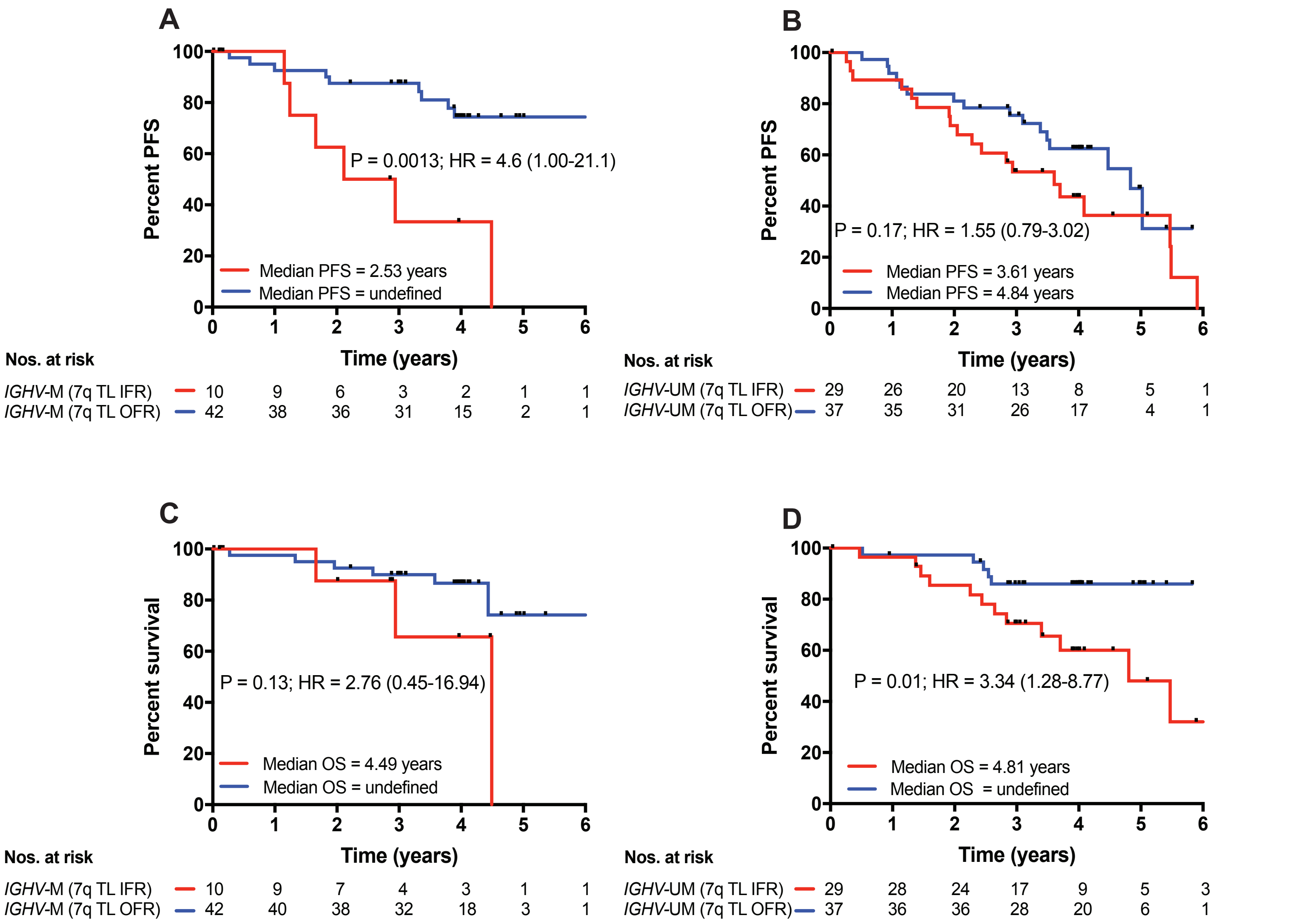

Supplementary Figure 4. The impact of telomere length in *IGHV*-mutated and *IGHV*-unmutated groups in FCR-treated patients. Dividing the *IGHV*-mutated and *IGHV*-unmutated prognostic groups according to telomere length revealed (A) a significant difference in PFS in the *IGHV*-mutated group with short telomere patients having shorter PFS. (B) Short telomere length in the *IGHV*-unmutated group showed a trend towards reduced PFS but this was not significant. In terms of OS, (C) the *IGHV*-mutated group showed showed a trend towards reduced OS but this was not significant and (D) *IGHV*-unmutated groups showed significantly reduced OS.

Supplementary Figure 4
